# IgA and IgG1 Specific to Vi Polysaccharide of *Salmonella* Typhi Correlate With Protection Status in a Typhoid Fever Controlled Human Infection Model

**DOI:** 10.3389/fimmu.2019.02582

**Published:** 2019-11-01

**Authors:** Lindsay C. Dahora, Celina Jin, Rachel L. Spreng, Frederick Feely, Ryan Mathura, Kelly E. Seaton, Lu Zhang, Jennifer Hill, Elizabeth Jones, S. Munir Alam, S. Moses Dennison, Andrew J. Pollard, Georgia D. Tomaras

**Affiliations:** ^1^Duke Human Vaccine Institute, Duke University, Durham, NC, United States; ^2^Department of Immunology, Duke University, Durham, NC, United States; ^3^Oxford Vaccine Group, Department of Paediatrics, The NIHR Oxford Biomedical Research Centre, University of Oxford, Oxford, United Kingdom; ^4^Department of Medicine, Duke University, Durham, NC, United States; ^5^Department of Surgery, Duke University, Durham, NC, United States; ^6^Department of Pathology, Duke University, Durham, NC, United States; ^7^Molecular Genetics and Microbiology, Duke University, Durham, NC, United States

**Keywords:** typhoid fever, *Salmonella* Typhi (*S*. Typhi), Vi polysaccharide, correlates of protection (CoP), humoral immunity, vaccination, antibody, avidity

## Abstract

Vaccination against *Salmonella* Typhi using the Vi capsular polysaccharide, a T-cell independent antigen, can protect from the development of typhoid fever. This implies that antibodies to Vi alone can protect in the absence of a T cell-mediated immune response; however, protective Vi antibodies have not been well-characterized. We hypothesized that variability in the biophysical properties of vaccine-elicited antibodies, including subclass distribution and avidity, may impact protective outcomes. To interrogate the relationship between antibody properties and protection against typhoid fever, we analyzed humoral responses from participants in a vaccine efficacy (VE) trial using a controlled human infection model (CHIM) who received either a purified Vi polysaccharide (Vi-PS) or Vi tetanus toxoid conjugate (Vi-TT) vaccine followed by oral challenge with live *S*. Typhi. We determined the avidity, overall magnitude, and vaccine-induced fold-change in magnitude from before immunization to day of challenge of Vi IgA and IgG subclass antibodies. Amongst those who received the Vi-PS vaccine, Vi IgA magnitude (FDR *p* = 0.01) and fold-change (FDR *p* = 0.02) were significantly higher in protected individuals compared with those individuals who developed disease (“diagnosed”). In the Vi-TT vaccine group, the responses of protected individuals had higher fold-change in Vi IgA (FDR *p* = 0.06) and higher Vi IgG1 avidity (FDR *p* = 0.058) than the diagnosed Vi-TT vaccinees, though these findings were not significant at *p* < 0.05. Overall, protective antibody signatures differed between the Vi-PS and Vi-TT vaccines, thus, we conclude that although the Vi-PS and Vi-TT vaccines were observed to have similar efficacies, these vaccines may protect through different mechanisms. These data will inform studies on mechanisms of protection against typhoid fever, including identification of antibody effector functions, as well as informing future vaccination strategies.

## Introduction

The bacterial pathogen *Salmonella enterica* serovar Typhi (*S*. Typhi) is the leading cause of enteric fever world-wide and is responsible for nearly 20 million infections and ~200,000 deaths annually. Infection predominantly affects travelers and citizens from low-income countries, where children under 5 years of age face the highest disease burden (1, 2). Historically, antibiotics have been the standard treatment for infection. However, antibiotic resistance among *S*. Typhi clones has dramatically increased in endemic regions including the emergence and spread of an extensively drug resistant (XDR) clone throughout Pakistan in 2018 (3, 4). This has sparked concern for continued spread of XDR *S*. Typhi internationally, therefore, vaccination efforts against typhoid fever have become the primary focus for prevention (5).

The live-attenuated Ty21a vaccine and the subunit Vi polysaccharide (Vi-PS) vaccine constitute two of the most widely used typhoid fever vaccines worldwide. Both Ty21a and Vi-PS exhibit moderate efficacy of ~50–55% in the first year (6, 7), but neither is suitable for use in infants and young children. Ty21a is limited to use in children over 5 years of age due to formulation in large oral capsules that are difficult to swallow. Vi-PS is non-immunogenic in children under 2 years of age (8, 9) due to the nature of the Vi polysaccharide as a T-cell independent antigen that does not engage the T cell pool or stimulate germinal center reactions in young children (9, 10). Conversely, the Vi polysaccharide capsule confers bacterial resistance to complement deposition and phagocytosis (11–13), allowing the bacterium to disseminate systemically; therefore, Vi remains a major target for vaccination efforts against typhoid fever.

In light of this, typhoid protein-polysaccharide conjugate vaccines (TCVs) have been developed which may improve immune responses in early childhood and help combat this illness (14). In particular, a Vi-tetanus toxoid (Vi-TT) conjugate vaccine, which has recently undergone WHO prequalification, has been shown to be more immunogenic in adults than the Vi-PS vaccine (15). Vi-TT was evaluated for efficacy in the recent Vaccines against *Salmonella* Typhi (VAST) trial, in which healthy adult volunteers were vaccinated with either Vi-PS or Vi-TT and then orally challenged with live *S*. Typhi bacteria. Following challenge, participants exhibiting a positive *S*. Typhi blood culture and/or prolonged fever of ≥38°C for ≥12 h were defined as diagnosed. Interestingly, the attack rate, defined as the proportion of participants diagnosed with typhoid fever, was similar between vaccine groups, with 37% of Vi-PS vaccinees being diagnosed with typhoid fever and 35% of Vi-TT vaccinees being diagnosed (15). The lack of increased efficacy of the conjugate vaccine compared with the purified polysaccharide vaccine highlights the need for determining and better understanding the characteristics which confer protective immunity to typhoid fever.

For many years, research on immune mechanisms of protection against typhoid fever have been greatly hindered due to *S*. Typhi restriction to human hosts (11). However, regardless of similar attack rates between the Vi-PS and Vi-TT vaccines, the VAST trial, which used a controlled human infection model (CHIM) to measure efficacy, provides a direct approach to investigate the human immune response and identify immunological correlates of protection against typhoid fever. Despite higher immunogenicity of the Vi-TT vaccine, a significant difference in Vi total IgG titers between diagnosed and protected individuals was only observed in the Vi-PS group (15). Moreover, when comparing IgG1, IgG2, and IgG3, only Vi IgG2 subclass titers predicted protection in the Vi-PS group. While there were no correlations with Vi IgG subclass titer and protection in the Vi-TT group, Vi-TT vaccinees reported less severe clinical symptoms and most diagnosed participants had positive bacteremia with no reported fever whereas a higher proportion of diagnosed Vi-PS vaccinees had both fever and bacteremia (15). Given these differences in immune response and patterns of protection, we hypothesized that variability in the biophysical properties of antibodies induced by the Vi-PS and Vi-TT vaccines, including antibody subclass distribution and avidity, may impact protective outcomes. The Vi-PS vaccine, which exhibits moderate efficacy over 3 years, demonstrates that antibodies against Vi can impart protection in the absence of a cell-mediated response (16, 17). However, the critical classes, characteristics, and functional mechanisms of these protective antibodies against typhoid fever have not been identified.

It is well-established that different isotypes (IgA vs. IgG) and subclasses (IgG1 vs. IgG3) of antibodies have different distributions throughout the body as well as different biophysical characteristics that greatly influence antibody affinity (18, 19). These characteristics also determine success in antigen binding, recruitment of effector cells, and subsequent antibody Fc-mediated effector functions (20). Here we evaluate the maturation level and magnitude of the circulating antibody response to the Vi polysaccharide of *S*. Typhi and determine whether these measures correlate with protection in a typhoid fever CHIM. Determining the nature of the vaccine-elicited antibody response including proportion of Ig subclasses and affinity of those antibodies may be important in further identifying which functional mechanisms play a role in protection. These insights on antibody biomarkers of protection will pave the way for advances in vaccine design toward more efficacious vaccines, allow comparative assessment of new Vi-conjugates, and inform the use of Vi-conjugate vaccines in new populations, with resulting improvements in health.

## Materials and Methods

### VAST Trial

The VAST clinical trial (Clinicaltrials.gov ID: NCT02324751) was conducted as previously described (15). Briefly, participants provided written informed consent to receive either a single-dose control meningococcal-conjugate vaccine, a Vi polysaccharide (Typhim, Vi-PS) vaccine, or a Vi-tetanus toxoid conjugate vaccine (Typbar TCV, Vi-TT) intramuscularly at day minus 28 (D-28) (pre-vaccination/baseline). At 4 weeks post-vaccination, day 0 (D0), participants were challenged with *S*. Typhi by oral ingestion and monitored for development of *S*. Typhi bacteremia or persistent fever ≥38°C for 12 or more h (diagnosed) or lack thereof (protected). Immune responses to vaccination were assessed in the Vi-PS (*n* = 35) and Vi-TT (*n* = 37) groups at 4 weeks post vaccination (D0) as well as 3 and 6 months post-challenge (D90, D180).

### Binding Antibody Multiplex Assay- Avidity Index (BAMA-AI)

The WHO international standard for Vi polysaccharide (*C. freundii*, NIBSC, UK, Product Code: 12/244) (21) was biotinylated by Innova Biosciences. Vi polysaccharide and tetanus toxoid binding assays were modified from the binding antibody multiplex assay (22, 23). Briefly, biotinylated Vi polysaccharide (ViBiot) was conjugated to neutravidin-coupled, magnetic, Luminex microspheres, and tetanus toxoid (Reagent Proteins, USA) was conjugated by amine coupling with EDC/NHS. Native Vi polysaccharide (nViPS) was conjugated to polystyrene microspheres using an adipic acid dihydrazine linker as previously described (24, 25). Antigen-coupled beads were incubated with diluted vaccinee serum or plasma, followed by incubation in PBS or dissociative pH = 3 sodium-citrate (CIT) buffer. Detection reagents include R-Phycoerythrin-conjugated affiniPure goat anti-human IgA, alpha chain specific (Jackson Immunoresearch, USA), mouse anti-human IgA1-Biot (Southern Biotech, USA), mouse anti-human IgA2-Biot (SouthernBiotech, USA), mouse anti-human IgG1 antibody (BioLegend, USA), mouse anti-Human IgG2 (Biolegend, USA), and mouse anti-Human IgG3 (Invitrogen, USA) followed by goat anti-Mouse IgG, Human ads-PE (Southern Biotech, USA). IgG4 Vi and TT levels were below the limit of detection (data not shown). Fluorescence intensity (FI) was collected using the Bio-Plex 200 Platform. Plots are representative of *n* = 2 technical and experimental replicates. Positive controls included mouse anti-Vi IgG1 monoclonal (lot 188L-8; Statens Serum Institute Diagnostica A/S, DK) and WHO International Standard for anti-typhoid capsular Vi polysaccharide human IgG (16/138 WHO typhoid IS, NIBSC, UK, Product Code: 16/138). Normal human serum (NHS, Sigma, USA) and typhoid seronegative serum samples were used as negative controls, and non-specific binding to beads was controlled by subtracting FI reading of blank beads. IgA isotype assays were performed on IgG depleted serum or plasma. Magnitude of response was multiplied by dilution factor. Fold-change was calculated as the ratio of magnitude at D0, D90, or D180 to baseline (D-28). For MFI below 100, MFI was truncated to 100 for magnitude and fold-change calculations due to noise range of the instrument. Preset criteria for positive vaccine response were: MFI^*^Dilution > 95th percentile of baseline (D-28), MFI > 100, and MFI^*^Dilution >3-fold over subject-specific baseline (D-28) MFI^*^Dilution. Avidity Index (AI), expressed as a percentage, was calculated as $AI={\left[\frac{FI-Bkgd\;\left(CIT\right)}{FI-Bkgd\;\left(PBS\right)}\right]}^{*}100$. Subclass specific standard curves were included in every assay by conjugating the same primary detection antibodies that were used to detect the vaccine samples, including anti-human Ig (A, G2, G3), to beads via amine coupling followed by titration of known concentrations of purified Ig and detection with goat anti-human kappa-Biot (Southern Biotech, USA) and streptavidin-PE (BD Pharmingen, USA). The subclass standard curves utilized the exact same capture antibody that was used to detect the subclass-specific Vi antibodies in vaccinee sera/plasma, to ensure that the capture of polyclonal vaccine antibodies had equal sensitivity to that of the purified Ig capture in the subclass standard curves. Antibody concentrations were reported from serum dilutions in the linear range of the assays. For the IgG1 subclass standard curve, the goat anti-human kappa was amine coupled to bead, followed by titration of purified IgG1, and detected with Mouse anti-Human IgG1 (BioLegend, USA) and Goat anti-Mouse IgG, Human ads-PE (SouthernBiotech, USA), to detect vaccine specific responses (22). The μg/ml concentration of typhoid specific antibodies were calculated for positive vaccine responders only (defined as MFI^*^dilution >95th percentile of the baseline (D-28), MFI > 100, and MFI^*^Dilution >3-fold over the subject-specific baseline).

### Human Isotyping Immunoassay

The total concentration of antibody within 16/138 WHO typhoid IS was quantified using the Bio-Plex Pro Human Isotyping Assay kit per manufacturer's instructions (26). Briefly, anti-human IgA, IgM, IgG1, IgG2, IgG3, and IgG4-conjugated magnetic microspheres were incubated with titrated 16/138 WHO typhoid IS serum followed by primary and secondary detection. Fluorescence intensity (FI) was collected using the Bio-Plex 3D instrument (Bio-Rad, USA). The isotype and subclass components of the international serum standard, 16/138 typhoid IS, were determined with this method (5.69 mg/ml IgG1; 6.84 IgG2; 0.57 IgG3; 0.58 IgG4; 1.38 IgM; 0.96 IgA mean mg/ml, of 6 technical replicate measurements). These concentration units were utilized to calculate antibody subclass concentration in μg/ml equivalents of 16/138 in vaccinee sera. The 16/138 Typhoid IS IgA equivalents are noted for the non-IgG depleted standard.

### BioLayer Interferometry (BLI)

Polyclonal IgG fractions from vaccinees were purified from serum and plasma by Protein G HP MultiTrap™ plates (GE Healthcare, USA) prior to analysis by BLI. Kinetics of polyclonal antibody responses to Vi polysaccharide were analyzed by BLI as previously described (27) with modifications. Briefly, nViPS (5 μg/mL) was immobilized to aminopropylsilane (APS) biosensors via hydrophobic interaction. nViPS loaded sensors were then washed with 10X Kinetics Buffer (ForteBio, USA) in order to coat unoccupied sensor area and minimize non-specific binding. A baseline time course was established in 1X Kinetics Buffer, and then Ag-loaded sensors were dipped into wells containing 100 μg/mL (in 1X Kinetics buffer) purified IgG from vaccine participants to monitor Ab association. The dissociation step was monitored by dipping polyclonal Ab-bound sensors back into the 1X Kinetics buffer wells used to collect the baseline. Non-specific interactions were subtracted out using parallel blank sensors that were also washed in 10X Kinetics Buffer and dipped into polyclonal samples. The subtracted binding curves were used to obtain antibody binding response (nm) and dissociation rates (k_d_). Avidity score was calculated based on [binding response (nm)/dissociation rate (s^−1^)].

### Statistical Analyses

Comparisons between diagnosis status or between vaccine groups (Vi-PS vs. Vi-TT) were conducted by non-parametric Wilcoxon rank-sum tests. Paired comparisons between time points were conducted using non-parametric Wilcoxon signed-rank tests. Comparisons between ViBiot and nViPS antigens for any measurement (magnitude, fold-change, avidity index) were conducted using non-parametric Spearman rank correlation. Multivariate analysis of all variables measured by BAMA and BLI was performed using Principle Components Analysis (PCA). Statistical analyses were performed using R statistical software (version 3.5.1; R Foundation for Statistical Computing, AT). All raw *p*-values were adjusted within isotype and antigen using the Benjamini-Hochberg method to account for multiple testing; adjusted *p*-values <0.05 were considered significant.

## Results

### IgA Dominates the Vaccine-Elicited Antibody Response to Vi Polysaccharide

**Figure 1:**
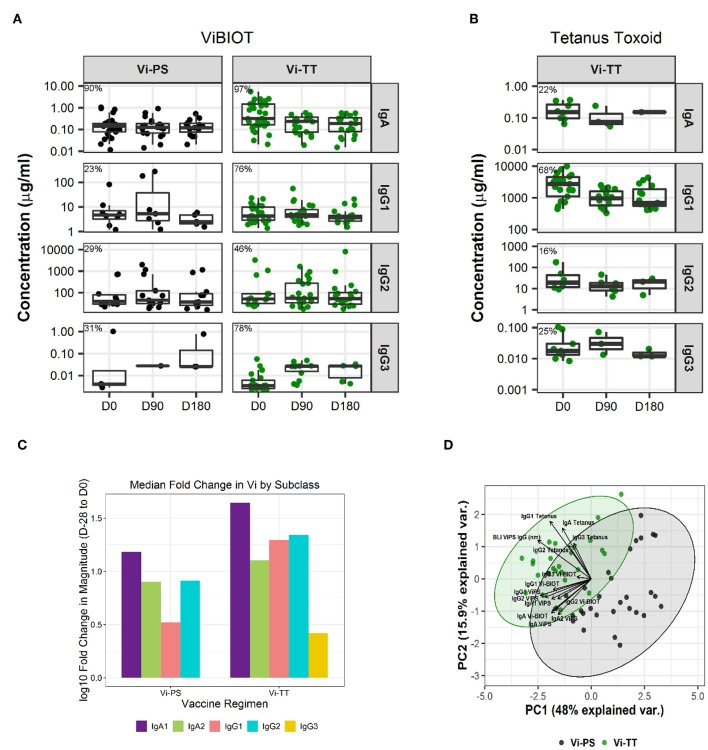
IgA dominates the vaccine-elicited antibody response to Vi polysaccharide. Concentration of antigen-specific, vaccine-induced IgA, IgG1, IgG2, and IgG3 to ViBIOT **(A)** and tetanus toxoid **(B)** by vaccine group, Vi-PS in black and Vi-TT in green, of positive vaccine responders only. Percent positive responders indicated post-vaccination at D0. Vi-PS vaccinees exhibited no vaccine-induced tetanus toxoid response. Data points are representative of *n* = 2 independent experiments (each with *n* = 2 technical replicates). Fold-change in magnitude of the response to Vi from Baseline to Day of Challenge across subclasses by vaccine group **(C)**. A principal components analysis with all tetanus and Vi responses included **(D)** with a scatter plot of the first (PC1) and second (PC2) principal components is shown. Each measurement from a Vi-PS (*n* = 35 participants) or a Vi-TT (*n* = 37 participants) vaccinee is represented by a black or green dot, respectively. Ellipses represent 95% confidence regions.

Assessment of subclass-specific Vi antibody concentrations in post-vaccination samples identified Vi IgG2 as the predominant subclass (antibody median concentration of ~10–20 μg/ml) (Figure 1A). However, after factoring in baseline responses (D-28) to include only positive vaccine responders, as previously defined in the methods section, Vi IgA antibodies were induced most in response to vaccination (D0). Specifically, Vi IgA1 antibodies exhibited the highest fold-change from baseline to day of challenge for both vaccine groups, followed by Vi IgG2 (Figure 1C, Table 1B). IgG3 responses were almost exclusively elicited in the Vi-TT vaccine group; however, the concentration was very low (Figure 1A). No antibody subclass was significantly boosted following oral challenge (D90, D180) with live *S*. Typhi (Figure 1A). As expected, the binding response to TT was only boosted in vaccinees who received Vi-TT and boosting was primarily in the IgG1 subclass (Figure 1B, Table 1A). Response rate and magnitude of IgA and IgG2 response to Vi by day of challenge (D0) was significantly higher in Vi-TT individuals; however, IgA and IgG2 median avidity was not significantly different between vaccine groups (Table 1B, Table 3) despite a broader range of IgA and IgG2 avidity in the Vi-TT group. To determine the relationship between the immune responses elicited by Vi-PS and Vi-TT vaccines, a principle components analysis (PCA) was conducted (Figure 1D). Variables include binding response magnitudes by BAMA and BLI for Vi and TT antigens across all subclasses: total IgA, IgA1, IgA2, IgG1, IgG2, IgG3, and total purified IgG (Figure 1D). At all the time points measured, IgG4 subclass antibodies to Vi and TT were below the limit of detection. Vi-TT vaccinees clustered away from Vi-PS vaccinees on the basis of higher tetanus and higher Vi antibody responses (Figure 1D), indicating that response to tetanus was a substantial driver of the differences observed between vaccine groups.

**TABLE 1A:** Antigen-specific magnitude by vaccine group at Day of challenge (D0).

**Subclass**	**Antigen**	**Vaccine arm**	**Response rate**	**Median mag^a^**	**Median mag^a^ range**
IgG1	ViBiot	Vi-PS	23% (8/35)	3.82E4	1.00E4–4.88E6
		Vi-TT	76% (28/37)	2.49E5	1.00E4–2.30E6
	TT	Vi-PS	0% (0/35)	2.60E5	5.30E4–5.72E6
		Vi-TT	68% (21/31)	8.51E6	5.21E5–5.30E7
IgG2	ViBiot	Vi-PS	29% (10/35)	6.25E4	4.00E3–5.44E6
		Vi-TT	46% (17/37)	1.59E5	4.00E3–2.35E8
	TT	Vi-PS	0% (0/35)	4.00E3	4.00E3–4.19E4
		Vi-TT	16% (6/37)	1.92E4	5.41E3–1.60E6
IgG3	ViBiot	Vi-PS	31% (11/35)	5.00E3	5.00E3–3.55 E6
		Vi-TT	78% (29/37)	1.31E4	5.00E3–3.17E5
	TT	Vi-PS	0% (0/34)	1.24E4	5.00E3–6.21E4
		Vi-TT	25% (9/36)	3.60E4	1.61E4–5.64E5
IgA	ViBiot	Vi-PS	88% (30/34)	4.71E5	2.43E4–5.78E6
		Vi-TT	97% (36/37)	1.05E6	9.78E4–1.86E7
	TT	Vi-PS	0% (0/35)	5.00E3	5.00E3–4.02E5
		Vi-TT	22% (8/37)	1.01E5	5.00E3–9.83E5

a Magnitude calculated as MFI^*^Dilution.

**TABLE 1B:** Antigen-Specific Responses by Vaccine Group at Day of Challenge (D0).

**Subclass**	**Antigen**	**Vaccine arm**	**Median mag log fold change^a^**	**AI^b^ median**	**AI^b^ range**
IgG1	ViBiot	Vi-PS	0.52	18.5	0–102
		Vi-TT	1.29	32	2–125
	TT	Vi-PS	0	0	0
		Vi-TT	1.17	37	11–70
IgG2	ViBiot	Vi-PS	0.91	27	2–74
		Vi-TT	1.34	47	9–91
	TT	Vi-PS	0	0	0
		Vi-TT	0.45	19.5	7–39
IgG3	ViBiot	Vi-PS	0	9	1–50
		Vi-TT	0.42	35	12–68
	TT	Vi-PS	0	0	0
		Vi-TT	0.64	74	16–88
IgA	ViBiot	Vi-PS	1.62	38.5	8–63
		Vi-TT	2.11	42	4–90
	TT	Vi-PS	0	0	0
		Vi-TT	0.78	53	31–70

a *Fold Change from Baseline (D-28) to Day of Challenge (D0)*.

b *AI, Avidity Index*.

### Higher Vi Polysaccharide-Specific IgA Magnitude and Fold-Change in Protected Vaccinees

**Figure 2:**
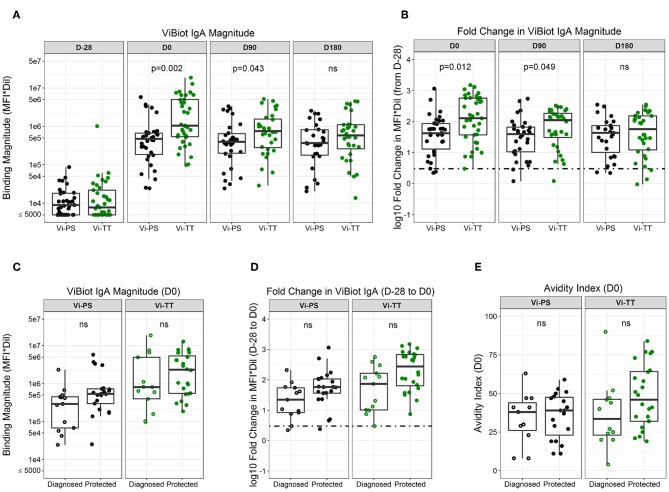
ViBIOT-specific total IgA magnitude, fold change, and avidity higher in protected individuals of both vaccine groups. ViBIOT-specific IgA magnitude **(A)** and fold change **(B)** by vaccine group over time. ViBIOT-specific IgA magnitude **(C)** and fold change **(D)** by diagnosed/protected outcome at day of challenge. ViBIOT IgA avidity index **(E)** by diagnosed/protected outcome at day of challenge. Diagnosed and protected individuals are represented by open and closed circles, respectively. Data points are representative of *n* = 2 independent experiments (each with *n* = 2 technical replicates). n.s indicates non-significant FDR-corrected *p* values.

To examine the question of whether pre-existing immunity impacted vaccine responsiveness, we evaluated the levels of baseline responses (D-28) across all antibody measurements in this study. The only antibody responses with a detectable measurement at baseline that showed a difference between responders and non-responders was IgA to tetanus (median MFI values of 563 and <100 at a 1:50 dilution, respectively). However, there was no difference in baseline responses between diagnosed vaccinees and protected vaccinees. Both the magnitude and fold-change from baseline (D-28) of ViBiot IgA were significantly higher in Vi-TT vaccinees at day of challenge (D0) and 3 months post-challenge (D90) when compared with Vi-PS; however, there was no significant difference between groups by 6 months post-challenge (D180) (Figures 2A,B, Supplementary Material). ViBiot-specific IgA magnitude was higher in protected compared with diagnosed Vi-PS vaccinees (Figure 2C, Table 2), and IgA fold-change was higher in protected compared with diagnosed Vi-TT vaccinees (Figure 2D, Table 2), however these observations were not statistically significant (Table 3, FDR *p* = 0.078, FDR *p* = 0.061). In addition, anti-Vi IgA avidity was slightly higher in protected individuals in the Vi-TT group, however this was not significant (Figure 2E, Table 2, FDR *p* = 0.231).

**TABLE 2:** Vi Polysaccharide responses by protection status at day of challenge (D0).

**Subclass**	**Vaccine arm**	**Protection status**	**Response rate**	**Median mag^a^**	**Median mag log fold change^b^**	**AI^c^ median**
IgG1	Vi-PS	Diagnosed	1/13 (8%)	3.54E4	0.43	10 (10–10)
		Protected	7/22 (32%)	4.09E4	0.61	27 (0–102)
	Vi-TT	Diagnosed	11/13 (85%)	2.63E5	1.09	22 (2–44)
		Protected	17/24 (71%)	2.38E5	1.33	46 (2–125)
IgG2	Vi-PS	Diagnosed	4/13 (31%)	8.62E4	0.87	25.5 (2–68)
		Protected	6/22 (27%)	4.98E4	0.98	35 (6–74)
	Vi-TT	Diagnosed	6/13 (46%)	1.59E5	1.34	26.5 (15–81)
		Protected	11/24 (46%)	1.77E5	1.38	50 (9–91)
IgG3	Vi-PS	Diagnosed	2/13 (15%)	5.00E3	0	3 (1–5)
		Protected	9/22 (41%)	5.00E3	0	18 (1–47)
	Vi-TT	Diagnosed	9/13 (69%)	1.09E4	0.34	35 (18–49)
		Protected	20/24 (83%)	1.36E4	0.44	37.5 (21–68)
IgA	Vi-PS	Diagnosed	11/13 (85%)	2.92E5	1.35	38 (8–63)
		Protected	19/21 (90%)	5.36E5	1.76	39 (11–59)
	Vi-TT	Diagnosed	12/13 (92%)	8.09E5	1.87	33.5 (4–90)
		Protected	24/24 (100%)	2.32E6	2.44	46 (19–84)

a Magnitude calculated as MFI^*^Dilution.

b *Fold Change from Baseline (D-28) to Day of Challenge (D0)*.

c *AI, Avidity Index*.

**TABLE 3A:** Primary statistical analysis of IgA responses to Vi at day of challenge (D0).

**Isotype**	**Measure**	**Analyte**	**Comparison**	**raw.p**	**FDR.p**
IgA	Magnitude	Vi-Biot	Vaccine	**0.0002**	**0.0017**
			Diagnosis Vi-PS	*0.0241*	0.0785
			Diagnosis Vi-TT	0.3528	0.4992
		nViPS	Vaccine	**0.0016**	**0.0088**
			Diagnosis Vi-PS	**0.0021**	**0.0098**
			Diagnosis Vi-TT	0.1159	0.2314
	Fold Change	Vi-Biot	Vaccine	**0.0028**	**0.0118**
			Diagnosis Vi-PS	*0.0503*	0.1348
			Diagnosis Vi-TT	*0.0179*	*0.0610*
		nViPS	Vaccine	**0.0028**	**0.0118**
			Diagnosis Vi-PS	*0.0058*	**0.0230**
			Diagnosis Vi-TT	*0.0397*	0.1190
	Avidity Index	Vi-Biot	Vaccine	0.1087	0.2314
			Diagnosis Vi-PS	0.7465	0.7998
			Diagnosis Vi-TT	0.1108	0.2314
		nViPS	Vaccine	0.1018	0.2314
			Diagnosis Vi-PS	0.4562	0.6051
			Diagnosis Vi-TT	0.2359	0.3539
IgA1	Magnitude	nViPS	Vaccine	**0.0011**	**0.0066**
			Diagnosis Vi-PS	0.1363	0.2324
			Diagnosis Vi-TT	0.2106	0.3224
	Fold Change	nViPS	Vaccine	**0.0012**	**0.0072**
			Diagnosis Vi-PS	0.1363	0.2324
			Diagnosis Vi-TT	*0.0362*	*0.1132*
	Avidity Index	nViPS	Vaccine	*0.0498*	0.1348
			Diagnosis Vi-PS	0.6409	0.7395
			Diagnosis Vi-TT	0.0503	0.1348
IgA2	Magnitude	nViPS	Vaccine	0.1278	0.2314
			Diagnosis Vi-PS	0.1027	0.2314
			Diagnosis Vi-TT	0.1660	0.2594
	Fold Change	nViPS	Vaccine	0.1278	0.2314
			Diagnosis Vi-PS	0.1027	0.2314
			Diagnosis Vi-TT	0.1660	0.2594
	Avidity Index	nViPS	Vaccine	0.5349	0.6800
			Diagnosis Vi-PS	0.7379	0.7998
			Diagnosis Vi-TT	0.0547	0.1416

*Raw p values < 0.005 and FDR-corrected p values < 0.05 bolded. Raw p values < 0.05 italicized. Vi-BIOT antigen data not collected for IgA1 and IgA2 subclasses*.

**TABLE 3B:** Primary statistical analysis of IgG responses to Vi at Day of challenge (D0).

**Isotype**	**Measure**	**Analyte**	**Comparison**	**raw.p**	**FDR.p**
IgG1	Magnitude	Vi-Biot	Vaccine	**0.00008**	**0.0010**
			Diagnosis Vi-PS	1.0000	1.0000
			Diagnosis Vi-TT	0.6717	0.7632
		nViPS	Vaccine	**0.00002**	**0.0006**
			Diagnosis Vi-PS	0.1272	0.2314
			Diagnosis Vi-TT	0.2908	0.4276
	Fold Change	Vi-Biot	Vaccine	**0.0004**	**0.0032**
			Diagnosis Vi-PS	0.7056	0.7899
			Diagnosis Vi-TT	0.7417	0.7998
		nViPS	Vaccine	**0.000002**	**0.0001**
			Diagnosis Vi-PS	0.0571	0.1429
			Diagnosis Vi-TT	0.9875	1.0000
	Avidity Index	Vi-Biot	Vaccine	0.1427	0.2365
			Diagnosis Vi-PS	Not tested	Not tested
			Diagnosis Vi-TT	*0.0154*	*0.0576*
		nViPS	Vaccine	**0.000003**	**0.0001**
			Diagnosis Vi-PS	0.9303	0.9691
			Diagnosis Vi-TT	0.6141	0.7197
IgG2	Magnitude	Vi-Biot	Vaccine	*0.0162*	*0.0577*
			Diagnosis Vi-PS	0.9866	1.0000
			Diagnosis Vi-TT	0.4223	0.5759
		nViPS	Vaccine	**0.0003**	**0.0025**
			Diagnosis Vi-PS	0.1210	0.2314
			Diagnosis Vi-TT	0.4598	0.6051
	Fold Change	Vi-Biot	Vaccine	**0.0021**	**0.0098**
			Diagnosis Vi-PS	0.4682	0.6055
			Diagnosis Vi-TT	0.8384	0.8856
		nViPS	Vaccine	**0.0006**	**0.0049**
			Diagnosis Vi-PS	0.1296	0.2314
			Diagnosis Vi-TT	0.3695	0.5132
	Avidity Index	Vi-Biot	Vaccine	0.1451	0.2365
			Diagnosis Vi-PS	Not tested	Not tested
			Diagnosis Vi-TT	0.1191	0.2314
IgG3	Magnitude	Vi-Biot	Vaccine	**0.000047**	**0.0007**
			Diagnosis Vi-PS	0.5950	0.7084
			Diagnosis Vi-TT	0.5758	0.7079
	Fold Change	Vi-Biot	Vaccine	**0.000045**	**0.0007**
			Diagnosis Vi-PS	0.5950	0.7084
			Diagnosis Vi-TT	0.5758	0.7079
	Avidity Index	Vi-Biot	Vaccine	**0.0007**	**0.0049**
			Diagnosis Vi-PS	NA	NA
			Diagnosis Vi-TT	0.2993	0.4316

*Raw p values < 0.005 and FDR-corrected p values < 0.05 bolded. Raw p values < 0.05 italicized. nViPS antigen data not collected for IgG2 avidity or the IgG3 subclass. “NA” indicates that data was not collected. “Not tested” indicates insufficient data points for statistical testing*.

**Figure 3:**
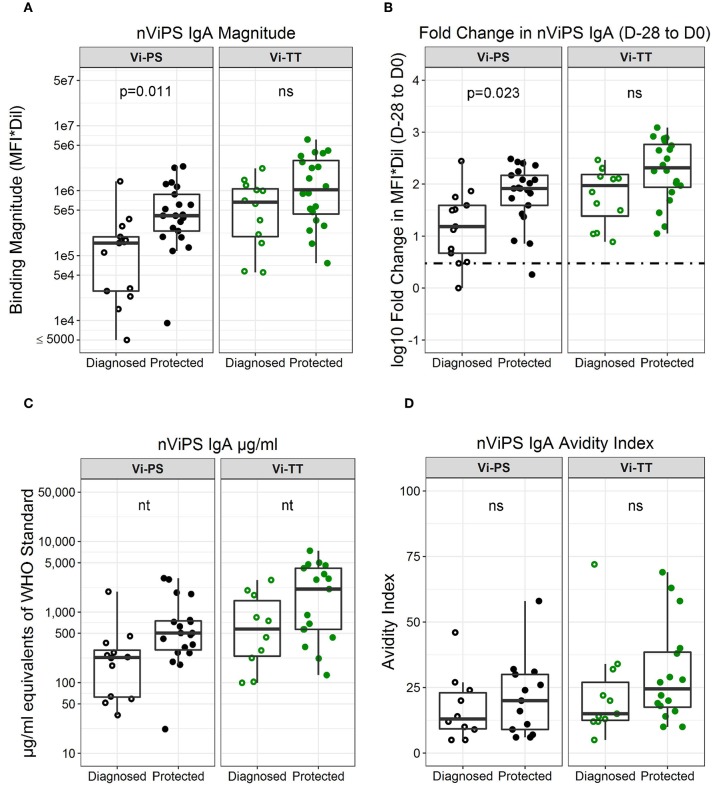
nViPS-specific total IgA magnitude, fold change, and avidity higher in protected individuals of both vaccine groups. nViPS-specific IgA magnitude **(A)** fold change **(B)** μg/mL equivalents of WHO typhoid IS 16/138 **(C)** and avidity index **(D)** by diagnosed/protected outcome at day of challenge. Data points are representative of *n* = 2 independent experiments (each with *n* = 2 technical replicates). n.s indicates non-significant FDR-corrected *p* values. nt indicates not tested for statistical significance.

To further characterize the antibody responses, we utilized two available forms of Vi polysaccharide (i.e., nViPS and ViBiot) with slightly different antigenicity profiles (Supplementary Material, Figure 1). Both magnitude (Figure 3A, Table 2, FDR *p* = 0.01) and fold-change (Figure 3B, *p* = 0.02) of nViPS IgA correlated with protection status in the Vi-PS vaccine group. Similar to ViBiot IgA, fold-change of nViPS IgA appeared higher in protected individuals of the Vi-TT vaccine group, however this was not statistically significant (Figure 3B, FDR *p* = 0.12). To determine whether there was a protective threshold concentration of Vi-specific IgA and to facilitate comparison across studies and trials, we calculated the concentration of Vi-specific IgA in μg/ml equivalents of the WHO typhoid Serum International Standard (NIBSC 16/138). The effect size of the difference in nViPS IgA antibody between protected individuals of the Vi-PS group (median = 504 μg/ml) compared with diagnosed (median = 227 μg/ml) was nearly 2-fold. In the Vi-TT group, the effect size of the difference in nViPS IgA between the protected Vi-TT group (median = 2,118 μg/ml) compared with diagnosed (median = 595 μg/ml) was 3.5-fold. However, there was no threshold above which 100% of individuals were protected (Figure 3C) suggesting that other immune mechanisms contribute to protection status. The maturation of the vaccine-elicited IgA response, as measured by IgA avidity index, was not significantly different between protected and diagnosed individuals for either vaccine group (Figure 3D).

### Vi IgA1 and IgA2 Is Higher in Protected Individuals

**Figure 4:**
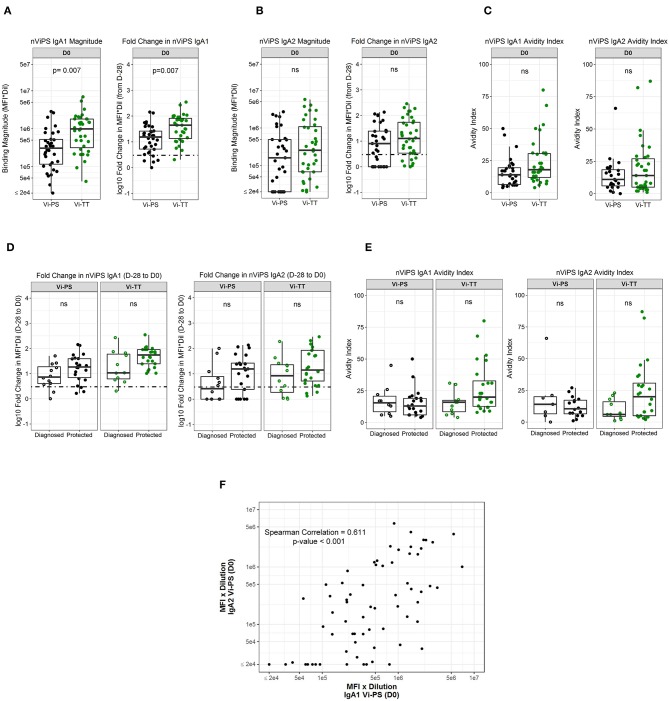
nViPS IgA1 and IgA2 fold change higher in protected individuals in both vaccine groups. nViPS-specific IgA1 magnitude and fold change **(A)** and nViPS-specific IgA2 magnitude and fold change **(B)** by vaccine group from baseline to day of challenge. nViPS IgA1 and IgA2 avidity index **(C)** by vaccine group at day of challenge. IgA1 and IgA2 fold change from baseline to day of challenge by protected/diagnosed status **(D)** and IgA1 and IgA2 avidity index at day of challenge **(E)** by protected/diagnosed status. Data points are representative of *n* = 2 independent experiments (each with *n* = 2 technical replicates). Spearman correlation between nViPS IgA1 and IgA2 magnitude **(F)** at day of challenge. n.s indicates non-significant FDR-corrected *p* values.

Since nViPS IgA correlated with protection in Vi-PS vaccinees and was also higher in protected Vi-TT vaccinees, though not statistically significant, we determined whether this potentially protective mechanism was driven by IgA1 or IgA2 subclass responses. IgA1 magnitude (FDR *p* = 0.0070) and fold-change (FDR *p* = 0.007) were significantly higher in the Vi-TT group at day of challenge (D0) (Figure 4A). In contrast, nViPS IgA2 magnitude and fold-change were higher in the Vi-TT group compared with the Vi-PS group, but were not significantly different (Figure 4B). While there was no difference in IgA2 avidity between the vaccine groups, nViPS IgA1 avidity was higher in the Vi-TT group, though not statistically significant (Figure 4C, Table 3, FDR *p* = 0.14). Fold-change in nViPS IgA1 and IgA2 was higher in protected individuals of both vaccine groups; however, there was no significant difference (Figure 4D). Neither IgA1 nor IgA2 avidity index was higher in protected individuals of the Vi-PS vaccine group; however, for Vi-TT vaccinees, both nViPS IgA1 (FDR *p* = 0.14) and IgA2 (FDR *p* = 0.14) avidity index were higher in protected individuals, though not statistically significant (Figure 4E). In order to determine whether individuals with high IgA1 responses were associated with high IgA2 responses, we conducted a Spearman correlation of IgA1 and IgA2 response magnitude (Figure 4F). There was a moderate correlation between IgA1 and IgA2 magnitude (Spearman correlation = 0.611, *p* < 0.001), but not all individuals with high IgA1 exhibited high IgA2 overall suggesting a greater degree of subclass specificity in the response to Vi.

### IgG1 Avidity to Vi Polysaccharide Is Higher in Protected Individuals

**Figure 5:**
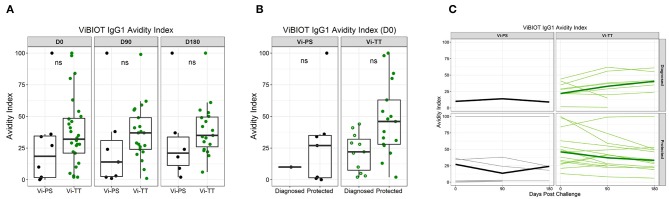
Vi polysaccharide IgG1 avidity higher in protected individuals. ViBIOT IgG1 avidity index at pH 3.0 in positive vaccine responders by vaccine group over time **(A)** and by diagnosed/protected outcome at day of challenge **(B)**. Avidity index in positive vaccine responses of anti-ViBIOT IgG1 over time **(C)** from 4 weeks post-vaccination (D0) to 6 months post-challenge (D180) by vaccine group and protection status. Bolded lines indicate median avidity index and faint lines indicate individual level data. Data points are representative of *n* = 2 independent experiments (each with *n* = 2 technical replicates). nt indicates not tested for statistical significance.

Given that the addition of protein carriers, such as tetanus toxoid, to a polysaccharide vaccine allows for T cell engagement and avidity maturation (14, 28), we determined whether the Vi-TT vaccine improved Vi antibody avidity, and whether avidity maturation was associated with protection. Overall, the median avidity to Vi polysaccharide was higher in the Vi-TT group across all subclasses (IgA, IgG1, IgG2, IgG3); however, the only significant difference in avidity between vaccine groups was for ViBiot IgG3 (Tables 1B, 3), where Vi IgG3 avidity was 4-fold higher in Vi-TT (median AI: 35%) vaccinees than Vi-PS vaccinees (median AI: 9%). However, IgG3 magnitudes in the Vi-PS group were very low. In the Vi-PS group, Vi IgG2 and IgG1 antibodies exhibited the highest avidity, with IgA1 and IgA2 in between, and IgG3 exhibiting the lowest avidity. In contrast, for the Vi-TT group, Vi IgG2 and IgG3 antibodies exhibited the highest avidity followed by IgG1, and the IgA subclasses exhibited the lowest (Table 1B). While there was no significant difference between Vi-PS and Vi-TT in ViBiot IgG1 avidity at any time point (Figure 5A), ViBiot IgG1 avidity was higher in protected individuals in the Vi-TT group, however this did not meet statistical significance at *p* < 0.05 (Figure 5B, FDR *p* = 0.058). The Vi-PS group was not tested for statistical differences among vaccine groups for IgG1 avidity due to low numbers of positive responders. To determine if the avidity of the vaccine-elicited IgG response further increased following oral challenge with live *S*. Typhi, we examined responses at 3 and 6 months post-challenge (D90, D180) compared to D0. There were no significant differences when compared with day of challenge (D0), of the median off-rate (s^−1^) of vaccinee purified polyclonal IgGs to nViPS by BLI analysis (Table 4). However, the median IgG1 avidity index modestly increased in the diagnosed group but not in the protected group at 3 and 6 months post-challenge (Figure 5C).

**TABLE 4:** Dissociation rates of purified polyclonal IgGs to nViPS.

**Days post challenge**	**Vaccine arm**	**Median Off-rate (s^**−1**^)**	**Range (s^**−1**^)**
D0	Vi-PS	1.44E-03	2.63E-02–1.00E-06
	Vi-TT	8.13E-04	9.01E-03–1.00E-06
D90	Vi-PS	1.28E-03	2.21E-03–1.00E-06
	Vi-TT	1.04E-03	4.36E-03–1.00E-06
D180	Vi-PS	2.12E-03	3.70E-03–2.65E-04
	Vi-TT	1.28E-03	2.87E-03–1.20E-04

### Vi-PS and Vi-TT Vaccines Elicit Differential Immune Responses Associated With Protection

**Figure 6:**
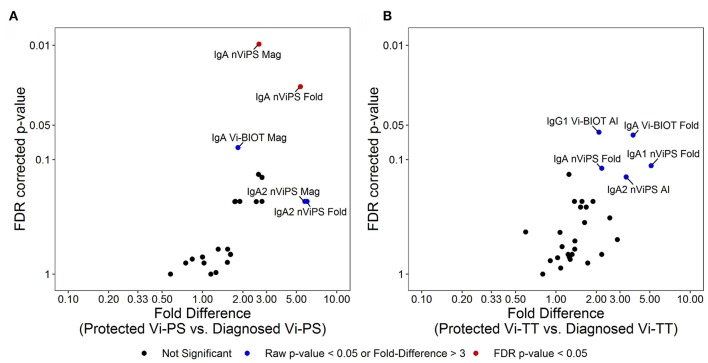
Different immune signatures of protection status for Vi-PS and Vi-TT. Variables in analysis of immune signatures between vaccine groups include magnitude, fold-change, and avidity of IgA1, IgA2, IgG1, IgG2, and IgG3 to Vi-Biot and nViPS. Volcano plot of FDR-corrected *p*-value and fold difference by diagnosed/protected outcome for Vi-PS vaccinees **(A)** and Vi-TT vaccinees **(B)**. Red dots represent FDR-corrected *p*-values < 0.05, blue dots represent raw *p*-values <0.05 or fold difference >3 (trending).

To better understand what factors are associated with protection in Vi-PS vs. Vi-TT vaccinees, we examined data collected across all antibody subclasses. In Vi-PS vaccinees, protection was significantly associated with Vi IgA magnitude and fold-change, and protected subjects had over 5-fold higher Vi IgA2 magnitude and fold-change compared with diagnosed vaccinees (Figure 6A). In Vi-TT vaccinees, there were no significant associations with protection, but distinct trends were observed for higher anti-Vi IgG1 avidity as well as total IgA fold-change, IgA1 fold change, and IgA2 avidity in protected individuals (Figure 6B).

## Discussion

Although moderately efficacious vaccines against *S*. Typhi have been licensed for decades, identification of mechanistic correlates of protection (mCoP), which could be used to improve vaccine design, have not been identified. This is because *S*. Typhi is a human restricted pathogen, thus limiting insights into the pathogenesis of typhoid fever and the host response (11). The establishment of a CHIM for the evaluation of the WHO prequalified Vi-tetanus tetanus (Vi-TT) conjugate vaccine has provided a platform with which to directly characterize vaccine-elicited Vi antibodies obtained from protected and diagnosed individuals. This is difficult to accomplish in field efficacy trials, as it requires post-vaccine blood samples being available from tens of thousands of participants and prolonged observation for cases to occur naturally. Following oral challenge with live *S*. Typhi, both the Vi-PS and Vi-TT vaccines exhibited similar attack rates at 37 and 35%, respectively, despite higher immunogenicity of the Vi-TT vaccine. In addition, a significant difference in anti-Vi IgG titers between diagnosed and protected individuals was only observed in the Vi-PS group, particularly for the IgG2 subclass, yet Vi-TT vaccinees exhibited attenuated clinical outcomes compared with Vi-PS vaccinees (15). Based on these data, since there are no currently known direct cell-mediated effector mechanisms generated by PS or conjugate vaccines, we hypothesized that the Vi-PS and Vi-TT vaccines may elicit antibody responses with distinct biophysical properties including subclass distribution and avidity that may influence protective outcomes.

In this study, we sought to evaluate the effect of the tetanus carrier protein on the quality of the immune response, which may play a role in affinity maturation and isotype switching of antibodies via engagement of the T cell pool. In addition, we aimed to identify characteristics of vaccine-elicited antibodies that correlated with protection in the CHIM study. To evaluate and compare host responses between vaccine groups and protection status, we characterized the antibody (IgA, IgA1, IgA2, IgG1, IgG2, and IgG3) response pre-vaccination, post-vaccination, and up to 6 months post-challenge. We observed that among antibody types tested, the highest concentration of antibody belonged to the IgG2 subclass, consistent with studies showing that IgG2 is the predominant IgG subclass response to polysaccharide antigens (29). However, IgA dominated the overall vaccine-elicited antibody response to Vi in both groups, with the largest fold-change in IgA1, when only positive responders were evaluated. This is consistent with results from one study that detected higher frequencies of IgA antibody secreting cells (ASCs) compared with IgG ASCs following vaccination with Vi-PS (30). In addition, vaccination with Vi-PS or another Vi-conjugate vaccine using *Pseudomonas aeruginosa* recombinant exoprotein A (Vi-rEPA) demonstrated that fold-change in total IgA was higher than total IgG, though this study did not further classify by antibody subclass (8). None of the subclasses examined were boosted in response to challenge with live *S*. Typhi bacteria. This may have been a result of challenging volunteers near the peak of their post-vaccination antibody response when antibody titers were very high. Instead, challenge likely induced immuno-dominant responses to alternative antigens such as LPS and flagellin as has been previously described, at least amongst those who developed clinical or microbiological evidence of infection (31).

In evaluating antibody correlates of protection against typhoid fever, we examined both antibody magnitude and fold-change from baseline, as well as antibody avidity to Vi antigen. We observed that magnitude (FDR *p* = 0.01) and fold-change (FDR *p* = 0.02) from baseline of nViPS IgA correlated with protection status at day of challenge in the Vi-PS vaccine group. We also noted higher ViBiot IgA fold-change in the Vi-TT group (FDR *p* = 0.06), though not statistically significant. Protected individuals in both vaccine groups exhibited 2–3.5-fold higher concentrations of Vi-specific IgA compared with diagnosed individuals (in μg/ml equivalents of the 16/138 Typhoid IS Standard). Notably, the 16/138 Typhoid IS standard was non-IgG depleted therefore, IgA equivalents may be understated due to antigen-specific competition by IgG. However, the IgA correlation with protection status suggests that Vi IgA may serve as a surrogate marker that predicts protection from disease following vaccination. Studies of other infections have reported antibody titer and fold-rise as potential non-mechanistic correlates of protection (nCoP) that may aid in development and evaluation of vaccines (32–34); however, these data may also be evidence of an underlying mCoP that has yet to be identified. The Vi polysaccharide capsule, as the main virulence factor of *S*. Typhi, limits complement deposition and neutrophil chemotaxis which are essential in the immune response against *S*. Typhimurium (11–13). The lack of these innate immune responses to *S*. Typhi are what ultimately allow the bacteria to establish systemic infection; therefore, pre-existing Vi IgA may provide protection against *S*. Typhi infection via Fc-dependent mechanisms. IgA may activate the complement cascade via the lectin pathway and exhibit bactericidal activity (35), or alternatively enhance neutrophil phagocytosis (ADNP), as neutrophils express the highest levels of FcαR among the phagocytic immune cells (20). Serum bactericidal activity and high affinity opsonophagocytic antibodies remain the accepted correlates of protection for meningococcal (36–38) and pneumococcal (36, 39, 40) polysaccharide conjugate vaccines, respectively. For typhoid fever, studies have shown enhanced opsonophagocytosis of live *S*. Typhi using post-vaccination sera from various typhoid fever vaccine constructs (41, 42), yet none have done so with the Vi-PS or Vi-TT vaccines.

Further analysis of Vi IgA separated by IgA1 and IgA2 revealed that Vi IgA2 levels were 6-fold higher in protected vs. susceptible Vi-PS vaccinees. IgA2 is present in higher concentration in the mucosa than in serum (43). As such, this may suggest a role for IgA2 in mediating a protective effect at the gut epithelium prior to bacterial entrance into intracellular niches within the lymphatic system. It may also suggest a functional role for Vi IgA2 in the serum, as many studies have shown that anti-polysaccharide IgA responses are associated primarily with IgA2 subclass unless conjugated to a protein carrier (44, 45). In the Vi-TT group, anti-Vi IgA1 rather than IgA2 fold-change was 5-fold higher in protected individuals. These diverging data suggest that although IgA appears to predict protection in both vaccine groups, either the functional mechanism of IgA-mediated protection differs or IgA1 and IgA2 play redundant roles. A previous study on the Vi-PS vaccine demonstrated that nearly 60% of Vi-specific antibody secreting cells (ASCs) from human peripheral blood expressed gut-homing marker α_4_β_7_ while nearly 80% expressed systemic homing marker L-selectin (30). Therefore, it is still unknown whether Vi-specific ASCs mediate protective function at the mucosal or systemic level. Additional studies looking at plasmablast phenotypes and probing the mucosal compartment, including saliva Ig, may help determine whether the serum antibody pool is recapitulated at the mucosal level and may help to further elucidate the role of IgA, the most abundant class of antibody in the mucosa.

Previous studies have reported that tetanus toxoid as a carrier protein produces higher avidity antibodies against pneumococcal and *Haemophilus influenzae* type b (Hib) polysaccharides when compared with other licensed carrier proteins such as meningococcal outer membrane protein complex (OPMC) or diphtheria toxoid (28, 46, 47). Interestingly, the Vi-TT vaccine did not significantly increase the avidity of the response for any subclass other than IgG3 (FDR *p* = 0.005). However, though not statistically significant, avidity of each antibody subclass was higher in Vi-TT vaccinees compared with Vi-PS vaccinees. Despite lower median avidity compared with its IgG subclass counterparts, ViBiot IgG1 avidity was higher in protected Vi-TT vaccinees compared with diagnosed, though not statistically significant (FDR *p* = 0.058). IgG1 subclass antibodies have greater affinity for FcɤRs compared with IgG2 (20), therefore, these data may suggest a role for IgG1 in Fc-mediated protection against typhoid fever. IgG3 antibodies, like IgG1, are also known to bind to FcɤRs with higher affinity than IgG2 and IgG4; however, despite exhibiting higher avidity to Vi in the Vi-TT group, the magnitude of the IgG3 response was very low. In addition to Vi IgG1 avidity, Vi IgA2 avidity was 3-fold higher in protected vs. diagnosed Vi-TT vaccinees. Interestingly, IgA2 exhibited the lowest median avidity in the Vi-TT group yet showed the largest difference between protected and diagnosed individuals. This finding, along with the Vi IgG1 avidity, illustrates how antibody-mediated protection may encompass not only affinity for antigen but also distribution in the correct locations throughout the body as well as ability to engage available effector cells.

Unlike the Vi-TT group, there were no trends with avidity and protection status in the Vi-PS vaccinees. The increase in avidity of serum antibodies, expressed by avidity index and antibody off-rate, in Vi-TT vaccinees suggests that the tetanus carrier protein did stimulate germinal center reactions and affinity maturation. However, the avidity index of Vi antibodies did not exceed 50% post-vaccination, indicating that the antibodies exhibited low to medium avidity at best, even with the conjugate vaccine. In fact, most antibodies elicited by conjugate vaccines have low affinity to their polysaccharide ligands. Nearly all isolated polysaccharide monoclonal antibodies have micromolar binding affinities which is orders of magnitude lower than typical anti-protein antibodies (48–50). For example, in this study the off-rate (k_d_) of purified polyclonal IgGs from vaccinated individuals was on the order of 1e^−3^ s^−1^, whereas malaria-naïve subjects receiving the malaria RTS,S vaccine develop antibodies to circumsporozoite protein (CSP) with off-rates on the order of 1e^−4^ s^−1^ or less, measured using the same BLI method (27). However, these CSP antibodies underwent affinity maturation over the course of three doses of vaccine, whereas Vi-TT and Vi-PS vaccines were single dose. Additional studies examining the phenotype and functional properties of the TT-specific T cell pool may shed light on the low level of affinity maturation observed in this trial. Similar to the magnitude of the response, the avidity of Vi antibodies, across all subclasses, did not increase post-challenge with live *S*. Typhi. This could suggest that boosting at later time points is required for increased affinity maturation to Vi.

Examining how the biophysical properties of Vi antibodies relates to protection status has revealed that the Vi-PS and the Vi-TT vaccines elicit different protective antibody signatures. Trends in the protected group of Vi-PS vaccinees are dominated by total IgA and IgA2 levels whereas in the Vi-TT group, protected individuals appear to exhibit a signature consistent with high IgA levels and higher avidity responses for IgA2 and IgG1. Interestingly, although both the Vi-TT and Vi-PS vaccines exhibited a similar overall efficacy using stringent diagnosis criteria, fewer Vi-TT diagnosed vaccinees reported severe symptoms and fewer exhibited fever ≥ 38°C (15). In the VAST study, diagnostic criteria included prolonged fever of ≥ 38°C, *S*. Typhi bacteremia, or both together. In the Vi-PS group, more participants exhibited both criteria, whereas in the Vi-TT group, many exhibited bacteremia without fever that may have self-resolved (15). In fact, in a *post-hoc* analysis using the field definition of typhoid fever, which requires both fever and positive bacteremia, the Vi-TT vaccine efficacy was 87.1% and for Vi-PS only 52.3% (15). These improved clinical manifestations seem to suggest that a combined IgA response with higher affinity IgA and IgG1 antibody may provide better protection and attenuate disease outcome. In a typhoid endemic setting where pre-existing immunity, including IgA and IgG1 is likely to be present, the further boost in antibody maturation by vaccination (i.e., increasing antibody avidity), may enhance the protective response by vaccination, with a more pronounced boost in the Vi-TT group. Overall, the improved immunological properties of the Vi-TT conjugate vaccine are (i) higher IgG and IgA responses, (ii) immune responses in children under 2 (51), (iii) induction of immunological memory, and (iv) different pattern of antibody responses as shown in our studies. Additional studies are needed to determine if any of these responses relate to differences of VE in the field.

The analyses in this study were limited by statistical and ethical factors. Firstly, sample sizes in the CHIM were small, with merely 13 diagnosed individuals per group, and analysis of only positive vaccine responders and correcting for multiple comparisons affected the statistical power of the study. Furthermore, in the context of the CHIM, participants were treated immediately after identification of positive bacteremia or prolonged fever. Therefore, this provides a model of infection which differs from field trials in endemic settings in which disease is detected, as only symptomatic patients present to healthcare settings. In addition, considerations such as challenge dose, timing of challenge, and challenge population may all differ from natural infection. Importantly, the study population in this CHIM was typhoid naïve adults, and therefore, validation of these results in the target population, children in typhoid endemic regions, will be critical. Finally, we observed modest differences in the antigenicity of ViBiot and nViPS antigens in which responses positively correlated yet differed in statistical outcome. We believe that non-targeted biotinylation of the nViPS molecule may result in epitope changes that impact antigenicity, therefore, careful consideration, and characterization of immunogens is necessary. Studies using newly generated human monoclonal antibodies could help to resolve the fine specificity of antibody forms to these antigens elicited by typhoid fever vaccines.

Despite these limitations, the findings of this study demonstrate the utility of CHIMs in understanding protective immune responses to human-restricted pathogens and suggest that higher magnitude and affinity IgA may be a potential target for induction by typhoid fever vaccine strategies. This study identifies Vi IgA as a biomarker of protective immunity against typhoid fever and quantifies concentration of Vi IgA in vaccinees using the 16/138 typhoid International Standard (NIBSC). This standard could be used to compare vaccine-induced IgA responses across studies of other TCVs currently in development, such as the Vi-CRM_197_ and Vi-diphtheria toxin TCVs (52, 53), aiding vaccine evaluation. Using standard measures to compare across studies will allow careful consideration of which TCVs to deploy for routine immunization in infants under 2 years of age. Future work is required to understand the functional role of Vi IgA and determine whether IgA represents a mechanistic correlate of protection or a surrogate marker of an underlying immune response. IgA is known to play a critical role in mucosal immunity, and as *S*. Typhi is an enteric pathogen, IgA may be providing protection at the site of infection. Further studies are needed to determine whether the mucosal antibody response recapitulates the systemic compartment. More importantly, determining the potential IgA Fc-mediated functions that correlate with protection as well as further characterizing the epitope specificities of protective antibodies will inform vaccine design for elicitation of a more targeted and protective immune response against typhoid fever.

## Data Availability Statement

The datasets generated for this study are available on request to the corresponding author.

## Ethics Statement

The studies involving human participants were reviewed and approved by The South Central Oxford A Ethics Committee (14/SC/1427). The patients/participants provided their written informed consent to participate in this study.

## Author Contributions

LD, AP, and GT conceived and designed the work. CJ, JH, EJ, and AP carried out the clinical trial. LD, FF, and RM acquired data for the work. LD, CJ, SD, RS, LZ, KS, SA, AP, and GT analyzed and interpreted the work. KS, SD, SA, and GT designed experimental procedures and supervised. RS and LZ performed statistical analysis. LD, AP, and GT wrote the manuscript. CJ, RS, KS, and SD edited the manuscript. All other authors reviewed and approved the final document.

### Conflict of Interest

AP is Chair of UK Dept. Health Social Care's (DHSC) Joint Committee on Vaccination & Immunization (JCVI) & the European Medicine Agency (EMA) scientific advisory group on vaccines, and is a member of the WHO's Strategic Advisory Group of Experts. The views expressed in this article do not necessarily represent the views of DHSC, JCVI, or WHO. The remaining authors declare that the research was conducted in the absence of any commercial or financial relationships that could be construed as a potential conflict of interest.

## nomenclature

Controlled human infection model (CHIM); Vaccines against *Salmonella* Typhi (VAST); typhoid conjugate vaccine (TCV).

## References

[1] Antillon M Warren JL Crawford FW Weinberger DM Kurum E Pak GD. The burden of typhoid fever in low- and middle-income countries: a meta-regression approach. PLoS Negl Trop Dis. (2017) 11:e0005376. 10.1371/journal.pntd.0005376 28241011 PMC5344533

[2] Mogasale V Maskery B Ochiai RL Lee JS Mogasale VV Ramani E. Burden of typhoid fever in low-income and middle-income countries: a systematic, literature-based update with risk-factor adjustment. Lancet Glob Health. (2014) 2:e570–80. 10.1016/S2214-109X(14)70301-8 25304633

[3] Klemm EJ Shakoor S Page AJ Qamar FN Judge K Saeed DK. Emergence of an extensively drug-resistant salmonella enterica serovar typhi clone harboring a promiscuous plasmid encoding resistance to fluoroquinolones and third-generation cephalosporins. MBio. (2018) 9:e00105–18. 10.1128/mBio.00105-18 29463654 PMC5821095

[4] Wong VK Baker S Pickard DJ Parkhill J Page AJ Feasey NA. Phylogeographical analysis of the dominant multidrug-resistant H58 clade of Salmonella Typhi identifies inter- and intracontinental transmission events. Nat Genet. (2015) 47:632–9. 10.1038/ng.3281 25961941 PMC4921243

[5] Andrews JR Qamar FN Charles RC Ryan ET. Extensively drug-resistant typhoid - are conjugate vaccines arriving just in time? N Engl J Med. (2018) 379:1493–5. 10.1056/NEJMp1803926 30332569

[6] Black RE Levine MM Ferreccio C Clements ML Lanata C Rooney J. Efficacy of one or two doses of Ty21a Salmonella typhi vaccine in enteric-coated capsules in a controlled field trial. Chilean Typhoid Committee. Vaccine. (1990) 8:81–4. 10.1016/0264-410X(90)90183-M 2180234

[7] Engels EA Falagas ME Lau J Bennish ML. Typhoid fever vaccines: a meta-analysis of studies on efficacy and toxicity. BMJ. (1998) 316:110–6. 10.1136/bmj.316.7125.110 9462316 PMC2665386

[8] Kossaczka Z Lin FY Ho VA Thuy NT Van Bay P Thanh TC. Safety and immunogenicity of Vi conjugate vaccines for typhoid fever in adults, teenagers, and 2- to 4-year-old children in Vietnam. Infect Immun. (1999) 67:5806–10. 10531232 10.1128/iai.67.11.5806-5810.1999 PMC96958

[9] Rijkers GT Sanders EA Breukels MA Zegers BJ. Infant B cell responses to polysaccharide determinants. Vaccine. (1998) 16:1396–400. 10.1016/S0264-410X(98)00098-X 9711778

[10] Weintraub A. Immunology of bacterial polysaccharide antigens. Carbohydr Res. (2003) 338:2539–47. 10.1016/j.carres.2003.07.008 14670715

[11] Dougan G Baker S. Salmonella enterica serovar Typhi and the pathogenesis of typhoid fever. Annu Rev Microbiol. (2014) 68:317–36. 10.1146/annurev-micro-091313-103739 25208300

[12] Wilson RP Winter SE Spees AM Winter MG Nishimori JH Sanchez JF. The Vi capsular polysaccharide prevents complement receptor 3-mediated clearance of Salmonella enterica serotype Typhi. Infect Immun. (2011) 79:830–7. 10.1128/IAI.00961-10 21098104 PMC3028862

[13] Wangdi T Lee CY Spees AM Yu C Kingsbury DD Winter SE. The Vi capsular polysaccharide enables Salmonella enterica serovar typhi to evade microbe-guided neutrophil chemotaxis. PLoS Pathog. (2014) 10:e1004306. 10.1371/journal.ppat.1004306 25101794 PMC4125291

[14] Micoli F Adamo R Costantino P. Protein carriers for glycoconjugate vaccines: history, selection criteria, characterization and new trends. Molecules. (2018) 23. 10.3390/molecules23061451 PMC6100388 29914046

[15] Jin C Gibani MM Moore M Juel HB Jones E Meiring J. Efficacy and immunogenicity of a Vi-tetanus toxoid conjugate vaccine in the prevention of typhoid fever using a controlled human infection model of Salmonella Typhi: a randomised controlled, phase 2b trial. Lancet. (2017) 390:2472–80. 10.1016/S0140-6736(17)32149-9 28965718 PMC5720597

[16] Klugman KP Koornhof HJ Robbins JB Le Cam NN. Immunogenicity, efficacy and serological correlate of protection of Salmonella typhi Vi capsular polysaccharide vaccine three years after immunization. Vaccine. (1996) 14:435–8. 10.1016/0264-410X(95)00186-5 8735556

[17] MacLennan CA. Antibodies and protection against invasive salmonella disease. Front Immunol. (2014) 5:635. 10.3389/fimmu.2014.00635 25566248 PMC4273658

[18] Schroeder HW Jr Cavacini L. Structure and function of immunoglobulins. J Allergy Clin Immunol. (2010) 125:S41–52. 10.1016/j.jaci.2009.09.046 20176268 PMC3670108

[19] Bruhns P Iannascoli B England P Mancardi DA Fernandez N Jorieux S. Specificity and affinity of human Fcgamma receptors and their polymorphic variants for human IgG subclasses. Blood. (2009) 113:3716–25. 10.1182/blood-2008-09-179754 19018092

[20] Gunn BM Alter G. Modulating antibody functionality in infectious disease and vaccination. Trends Mol Med. (2016) 22:969–82. 10.1016/j.molmed.2016.09.002 27756530 PMC8767654

[21] Gao F Swann C Rigsby P Rijpkema S Lockyer K Logan A. Evaluation of two WHO first international standards for Vi polysaccharide from Citrobacter freundii and Salmonella enterica subspecies enterica serovar Typhi. Biologicals. (2019) 57:34–45. 10.1016/j.biologicals.2018.11.004 30502020

[22] Yates NL Liao HX Fong Y deCamp A Vandergrift NA Williams WT. Vaccine-induced Env V1-V2 IgG3 correlates with lower HIV-1 infection risk and declines soon after vaccination. Sci Transl Med. (2014) 6:228ra239. 10.1126/scitranslmed.3007730 24648342 PMC4116665

[23] Tomaras GD Yates NL Liu P Qin L Fouda GG Chavez LL Decamp AC. Initial B-cell responses to transmitted human immunodeficiency virus type 1: virion-binding immunoglobulin M (IgM) and IgG antibodies followed by plasma anti-gp41 antibodies with ineffective control of initial viremia. J Virol. (2008) 82:12449–63. 10.1128/JVI.01708-08 18842730 PMC2593361

[24] Biagini RE Schlottmann SA Sammons DL Smith JP Snawder JC Striley CA. Method for simultaneous measurement of antibodies to 23 pneumococcal capsular polysaccharides. Clin Diagn Lab Immunol. (2003) 10:744–50. 10.1128/CDLI.10.5.744-750.2003 12965898 PMC193885

[25] Schlottmann SA Jain N Chirmule N Esser MT. A novel chemistry for conjugating pneumococcal polysaccharides to Luminex microspheres. J Immunol Methods. (2006) 309:75–85. 10.1016/j.jim.2005.11.019 16448665

[26] Yates NL Stacey AR Nolen TL Vandergrift NA Moody MA Montefiori DC. HIV-1 gp41 envelope IgA is frequently elicited after transmission but has an initial short response half-life. Mucosal Immunol. (2013) 6:692–703. 10.1038/mi.2012.107 23299618 PMC3663876

[27] Dennison SM Reichartz M Seaton KE Dutta S Wille-Reece U Hill AVS. Qualified biolayer interferometry avidity measurements distinguish the heterogeneity of antibody interactions with plasmodium falciparum circumsporozoite protein antigens. J Immunol. (2018) 201:1315–26. 10.4049/jimmunol.1800323 30006374 PMC6077849

[28] Pichichero ME. Protein carriers of conjugate vaccines: characteristics, development, and clinical trials. Hum Vaccin Immunother. (2013) 9:2505–23. 10.4161/hv.26109 23955057 PMC4162048

[29] Vidarsson G Dekkers G Rispens T. IgG subclasses and allotypes: from structure to effector functions. Front Immunol. (2014) 5:520. 10.3389/fimmu.2014.00520 25368619 PMC4202688

[30] Kantele A Pakkanen SH Karttunen R Kantele JM. Head-to-head comparison of humoral immune responses to Vi capsular polysaccharide and Salmonella Typhi Ty21a typhoid vaccines–a randomized trial. PLoS ONE. (2013) 8:e60583. 10.1371/journal.pone.0060583 23593253 PMC3620468

[31] Waddington CS Darton TC Jones C Haworth K Peters A John T. An outpatient, ambulant-design, controlled human infection model using escalating doses of Salmonella Typhi challenge delivered in sodium bicarbonate solution. Clin Infect Dis. (2014) 58:1230–40. 10.1093/cid/ciu078 24519873 PMC3982839

[32] Gilbert PB Gabriel EE Miao X Li X Su SC Parrino J. Fold rise in antibody titers by measured by glycoprotein-based enzyme-linked immunosorbent assay is an excellent correlate of protection for a herpes zoster vaccine, demonstrated via the vaccine efficacy curve. J Infect Dis. (2014) 210:1573–81. 10.1093/infdis/jiu279 24823623 PMC4215071

[33] Bothamley GH. Epitope-specific antibody levels in tuberculosis: biomarkers of protection, disease, and response to treatment. Front Immunol. (2014) 5:243. 10.3389/fimmu.2014.00243 24917863 PMC4040437

[34] Jacobsen H Rajendran M Choi A Sjursen H Brokstad KA Cox RJ. Influenza virus hemagglutinin stalk-specific antibodies in human serum are a surrogate marker for *in vivo* protection in a serum transfer mouse challenge model. MBio. (2017) 8:e01463–17. 10.1128/mBio.01463-17 28928215 PMC5605943

[35] Roos A Bouwman LH van Gijlswijk-Janssen DJ Faber-Krol MC Stahl GL Daha MR. Human IgA activates the complement system via the mannan-binding lectin pathway. J Immunol. (2001) 167:2861–8. 10.4049/jimmunol.167.5.2861 11509633

[36] Plotkin SA. Correlates of protection induced by vaccination. Clin Vaccine Immunol. (2010) 17:1055–65. 10.1128/CVI.00131-10 20463105 PMC2897268

[37] Boyd MA Tennant SM Saague VA Simon R Muhsen K Ramachandran G. Serum bactericidal assays to evaluate typhoidal and nontyphoidal Salmonella vaccines. Clin Vaccine Immunol. (2014) 21:712–21. 10.1128/CVI.00115-14 24623629 PMC4018884

[38] Borrow R Balmer P Miller E. Meningococcal surrogates of protection–serum bactericidal antibody activity. Vaccine. (2005) 23:2222–7. 10.1016/j.vaccine.2005.01.051 15755600

[39] Lee LH Frasch CE Falk LA Klein DL Deal CD. Correlates of immunity for pneumococcal conjugate vaccines. Vaccine. (2003) 21:2190–6. 10.1016/S0264-410X(03)00025-2 12706710

[40] Romero-Steiner S Musher DM Cetron MS Pais LB Groover JE Fiore AE. Reduction in functional antibody activity against Streptococcus pneumoniae in vaccinated elderly individuals highly correlates with decreased IgG antibody avidity. Clin Infect Dis. (1999) 29:281–8. 10.1086/520200 10476727

[41] Wahid R Zafar SJ McArthur MA Pasetti MF Levine MM Sztein MB. Live oral Salmonella enterica serovar Typhi vaccines Ty21a and CVD 909 induce opsonophagocytic functional antibodies in humans that cross-react with S. Paratyphi A and S. Paratyphi B. Clin Vaccine Immunol. (2014) 21:427–34. 10.1128/CVI.00786-13 24429069 PMC3957674

[42] Lindow JC Fimlaid KA Bunn JY Kirkpatrick BD. Antibodies in action: role of human opsonins in killing Salmonella enterica serovar Typhi. Infect Immun. (2011) 79:3188–94. 10.1128/IAI.05081-11 21628517 PMC3147595

[43] Delacroix DL Dive C Rambaud JC Vaerman JP. IgA subclasses in various secretions and in serum. Immunology. (1982) 47:383–5. 7118169 PMC1555453

[44] Lue C Tarkowski A Mestecky J. Systemic immunization with pneumococcal polysaccharide vaccine induces a predominant IgA2 response of peripheral blood lymphocytes and increases of both serum and secretory anti-pneumococcal antibodies. J Immunol. (1988) 140:3793–800. 3372993

[45] Tarkowski A Lue C Moldoveanu Z Kiyono H McGhee JR Mestecky J. Immunization of humans with polysaccharide vaccines induces systemic, predominantly polymeric IgA2-subclass antibody responses. J Immunol. (1990) 144:3770–8. 2110213

[46] Schlesinger Y Granoff DM. Avidity and bactericidal activity of antibody elicited by different Haemophilus influenzae type b conjugate vaccines. The Vaccine Study Group. JAMA. (1992) 267:1489–94. 10.1001/jama.267.11.1489 1538539

[47] Anttila M Eskola J Ahman H Kayhty H. Differences in the avidity of antibodies evoked by four different pneumococcal conjugate vaccines in early childhood. Vaccine. (1999) 17:1970–7. 10.1016/S0264-410X(98)00458-7 10217596

[48] Cygler M Rose DR Bundle DR. Recognition of a cell-surface oligosaccharide of pathogenic Salmonella by an antibody Fab fragment. Science. (1991) 253:442–5. 10.1126/science.1713710 1713710

[49] Harris SL Fernsten P. Thermodynamics and density of binding of a panel of antibodies to high-molecular-weight capsular polysaccharides. Clin Vaccine Immunol. (2009) 16:37–42. 10.1128/CVI.00290-08 19005020 PMC2620655

[50] Haji-Ghassemi O Blackler RJ Martin Young N Evans SV. Antibody recognition of carbohydrate epitopesdagger. Glycobiology. (2015) 25:920–52. 10.1093/glycob/cwv037 26033938

[51] Laurens MB Sirima SB Rotrosen ET Siribie M Tiono A Ouedraogo A. A phase II, randomized, double-blind, controlled safety and immunogenicity trial of typhoid conjugate vaccine in children under 2 years of age in ouagadougou, Burkina Faso: A Methods Paper. Clin Infect Dis. (2019) 68:S59–66. 10.1093/cid/ciy1104 30845330 PMC6405275

[52] Bhutta ZA Capeding MR Bavdekar A Marchetti E Ariff S Soofi SB. Immunogenicity and safety of the Vi-CRM197 conjugate vaccine against typhoid fever in adults, children, and infants in south and southeast Asia: results from two randomised, observer-blind, age de-escalation, phase 2 trials. Lancet Infect Dis. (2014) 14:119–29. 10.1016/S1473-3099(13)70241-X 24290843

[53] Meiring JE Gibani M Ty VACCMG. The Typhoid Vaccine Acceleration Consortium (TyVAC): Vaccine Effectiveness Study Designs: Accelerating the Introduction of Typhoid Conjugate Vaccines and Reducing The Global Burden of Enteric Fever. Report from a meeting held on 26-27 October 2016, Oxford: UK (2017). 10.1016/j.vaccine.2017.08.001 28802757

