# Supplementary material for: IgA and IgG1 Specific to Vi Polysaccharide of Salmonella Typhi Correlate With Protection Status in a Typhoid Fever Controlled Human Infection Model
Source: Front Immunol. 2019 Nov 1;10:2582. doi: 10.3389/fimmu.2019.02582 (PMC6852708; doi:10.3389/fimmu.2019.02582)
Supplement: Supplementary file 1 [file Data_Sheet_1.docx]

**Supplementary Material**

**SOM Table 1. Secondary Statistical Analysis of Responses to Vi and tetanus toxoid**

| **Isotype** | | **Analyte** | **Measure** | **Comparison** | **Visit Date** | **raw.p** |
| --- | --- | --- | --- | --- | --- | --- |
| IgA | Vi-Biot | | Magnitude | Vaccine | D90 | *0.0427* |
|  |  |  |  | Vaccine | D180 | 0.2528 |
|  |  |  | Fold Change | Vaccine | D90 | *0.0489* |
|  |  |  |  | Vaccine | D180 | 0.4185 |
|  |  |  | Avidity Index | Vaccine | D90 | 0.3624 |
|  |  |  |  | Vaccine | D180 | 0.2867 |
|  | tetanus toxoid | | Magnitude | Vaccine | D0 | **3.13E-09** |
|  |  |  |  | Vaccine | D90 | **2.95E-06** |
|  |  |  |  | Vaccine | D180 | **0.0010** |
|  |  |  |  | Diagnosis Vi-PS | D0 | 0.8256 |
|  |  |  |  | Diagnosis Vi-TT | D0 | 0.8139 |
|  |  |  | Fold Change | Vaccine | D0 | **7.02E-13** |
|  |  |  |  | Vaccine | D90 | **2.61E-10** |
|  |  |  |  | Vaccine | D180 | **1.15E-05** |
|  |  |  |  | Diagnosis Vi-PS | D0 | 0.9714 |
|  |  |  |  | Diagnosis Vi-TT | D0 | 0.1406 |
| IgG1 | Vi-Biot | | Magnitude | Vaccine | D90 | **0.0006** |
|  |  |  |  | Vaccine | D180 | **0.0009** |
|  |  |  | Fold Change | Vaccine | D90 | **0.0012** |
|  |  |  |  | Vaccine | D180 | **0.0038** |
|  |  |  | Avidity Index | Vaccine | D90 | 0.1173 |
|  |  |  |  | Vaccine | D180 | 0.1814 |
|  | tetanus toxoid | | Magnitude | Vaccine | D0 | **7.14E-11** |
|  |  |  |  | Vaccine | D90 | **7.23E-08** |
|  |  |  |  | Vaccine | D180 | **2.04E-06** |
|  |  |  |  | Diagnosis Vi-PS | D0 | 0.1651 |
|  |  |  |  | Diagnosis Vi-TT | D0 | 0.1045 |
|  |  |  | Fold Change | Vaccine | D0 | **1.49E-11** |
|  |  |  |  | Vaccine | D90 | **4.93E-09** |
|  |  |  |  | Vaccine | D180 | **2.02E-07** |
|  |  |  |  | Diagnosis Vi-PS | D0 | 0.2509 |
|  |  |  |  | Diagnosis Vi-TT | D0 | 0.9173 |
| IgG2 | Vi-Biot | | Magnitude | Vaccine | D90 | *0.0484* |
|  |  |  |  | Vaccine | D180 | 0.3135 |
|  |  |  | Fold Change | Vaccine | D90 | *0.0143* |
|  |  |  |  | Vaccine | D180 | 0.1020 |
|  |  |  | Avidity Index | Vaccine | D90 | 0.6324 |
|  |  |  |  | Vaccine | D180 | 0.8128 |
|  | tetanus toxoid | | Magnitude | Vaccine | D0 | **7.82E-08** |
|  |  |  |  | Vaccine | D90 | **0.0004** |
|  |  |  |  | Vaccine | D180 | *0.0073* |
|  |  |  |  | Diagnosis Vi-PS | D0 | 0.1742 |
|  |  |  |  | Diagnosis Vi-TT | D0 | 0.2626 |
|  |  |  | Fold Change | Vaccine | D0 | **1.99E-12** |
|  |  |  |  | Vaccine | D90 | **2.35E-08** |
|  |  |  |  | Vaccine | D180 | **2.04E-05** |
|  |  |  |  | Diagnosis Vi-PS | D0 | *0.0206* |
|  |  |  |  | Diagnosis Vi-TT | D0 | 0.9375 |
| IgG3 | Vi-Biot | | Magnitude | Vaccine | D90 | **7.47E-06** |
|  |  |  |  | Vaccine | D180 | **1.94E-05** |
|  |  |  | Fold Change | Vaccine | D90 | **6.05E-06** |
|  |  |  |  | Vaccine | D180 | **1.66E-05** |
|  |  |  | Avidity Index | Vaccine | D90 | 0.0819 |
|  |  |  |  | Vaccine | D180 | NA |
|  | tetanus toxoid | | Magnitude | Vaccine | D0 | **5.13E-07** |
|  |  |  |  | Vaccine | D90 | **0.0004** |
|  |  |  |  | Vaccine | D180 | *0.0135* |
|  |  |  |  | Diagnosis Vi-PS | D0 | 0.1581 |
|  |  |  |  | Diagnosis Vi-TT | D0 | 0.2264 |
|  |  |  | Fold Change | Vaccine | D0 | **9.59E-12** |
|  |  |  |  | Vaccine | D90 | **1.22E-10** |
|  |  |  |  | Vaccine | D180 | **4.58E-07** |
|  |  |  |  | Diagnosis Vi-PS | D0 | *0.0359* |
|  |  |  |  | Diagnosis Vi-TT | D0 | 0.8933 |

Raw p values <0.005 **bolded** and raw p values <0.05 *italicized.* Avidity Index for tetanus toxoid not tested.

**SOM Figure 1A**

**
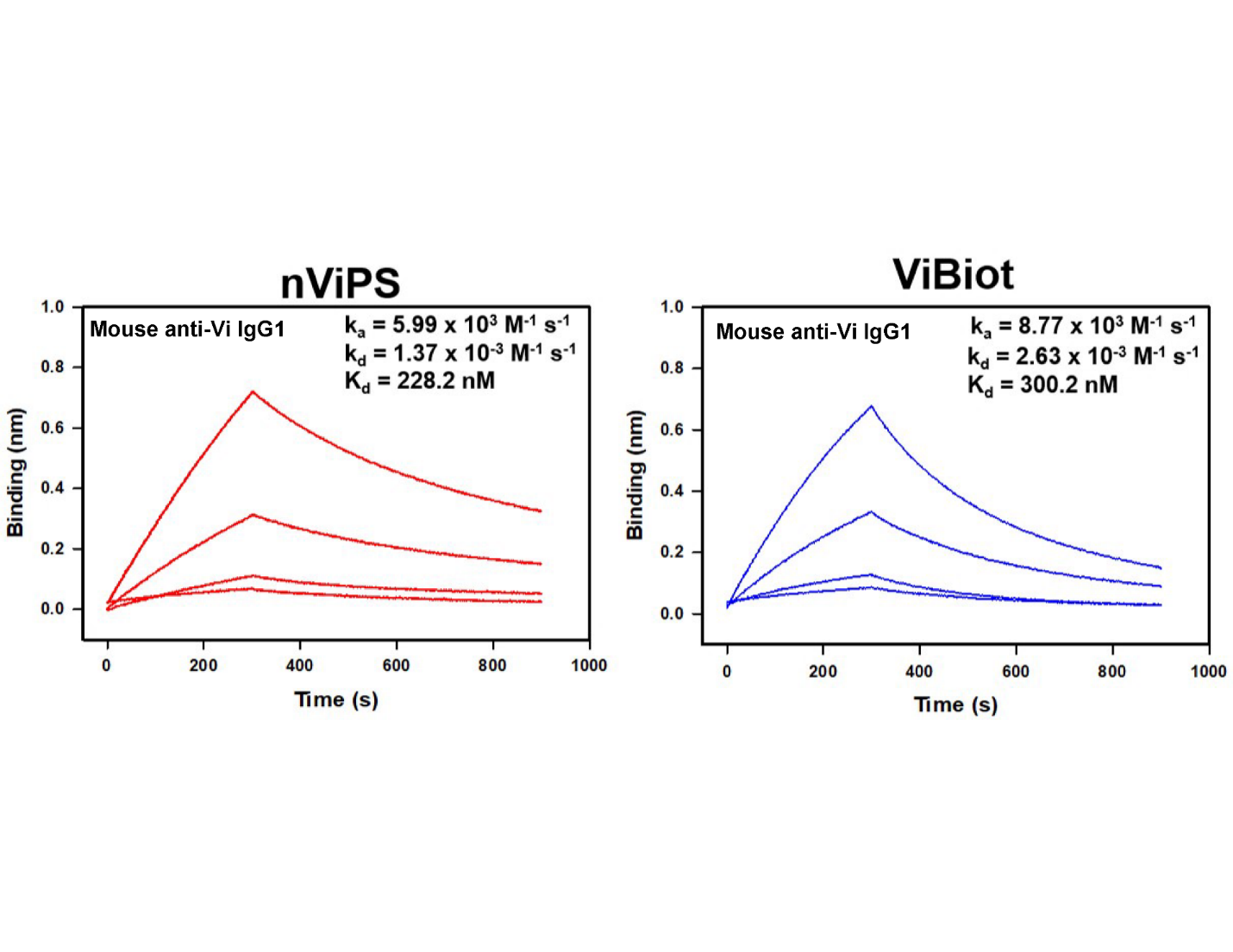
**

**SOM Figure 1B**


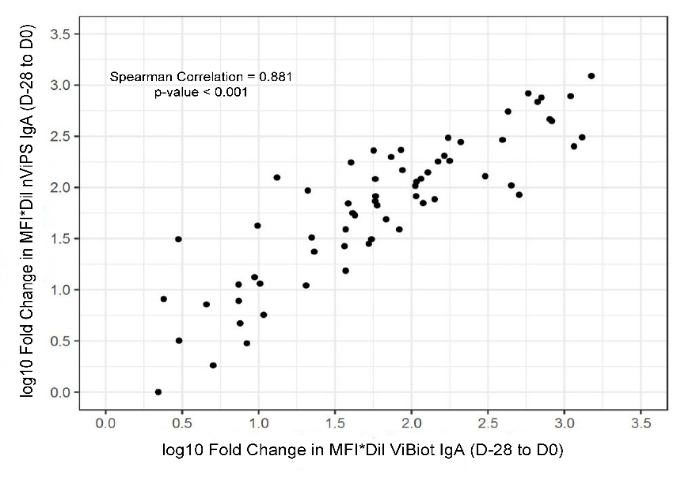

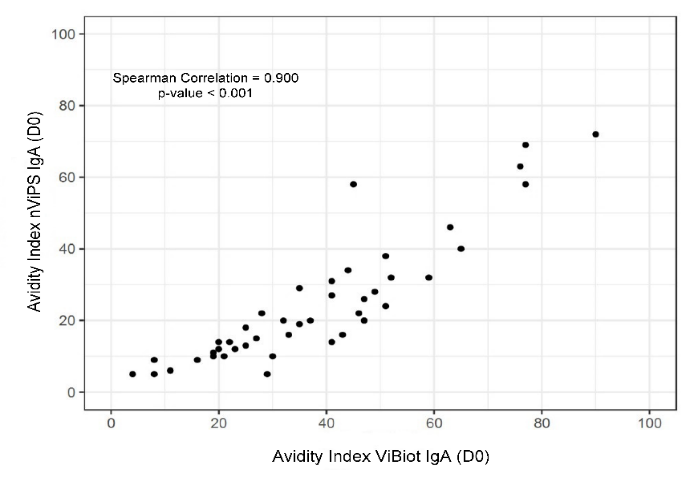


**SOM Figure 1. nViPS and ViBiot exhibit slight differences in antigenicity. (A)** Specific binding of mouse anti-human IgG1 (188L-8, SSI Diagnostica) monoclonal antibody (starting at 20 µg/ml titrated down 2-fold) to nViPS and ViBiot antigenic forms. **(B)** Spearman correlation for Vi IgA isotype antibody responses to nViPS and ViBiot for fold-change from baseline to day of challenge and avidity index.
